# Effect of Barodenervation on Cardiovascular Responses Elicited from the Hypothalamic Arcuate Nucleus of the Rat

**DOI:** 10.1371/journal.pone.0053111

**Published:** 2012-12-27

**Authors:** Tetsuya Kawabe, Kazumi Kawabe, Hreday N. Sapru

**Affiliations:** Department of Neurological Surgery, University of Medicine and Dentistry of New Jersey- New Jersey Medical School, Newark, New Jersey, United States of America; Hosptial Infantil Universitario Niño Jesús, CIBEROBN, Spain

## Abstract

We have previously reported that chemical stimulation of the hypothalamic arcuate nucleus (ARCN) in the rat elicited increases as well as decreases in blood pressure (BP) and sympathetic nerve activity (SNA). The type of response elicited from the ARCN (i.e., increase or decrease in BP and SNA) depended on the level of baroreceptor activity which, in turn, was determined by baseline BP in rats with intact baroreceptors. Based on this information, it was hypothesized that baroreceptor unloading may play a role in the type of response elicited from the ARCN. Therefore, the effect of barodenervation on the ARCN-induced cardiovascular and sympathetic responses and the neurotransmitters in the hypothalamic paraventricular nucleus (PVN) mediating the excitatory responses elicited from the ARCN were investigated in urethane-anesthetized adult male Wistar rats. Bilateral barodenervation converted decreases in mean arterial pressure (MAP) and greater splanchnic nerve activity (GSNA) elicited by chemical stimulation of the ARCN with microinjections of N-methyl-D-aspartic acid to increases in MAP and GSNA and exaggerated the increases in heart rate (HR). Combined microinjections of NBQX and D-AP7 (ionotropic glutamate receptor antagonists) into the PVN in barodenervated rats converted increases in MAP and GSNA elicited by the ARCN stimulation to decreases in MAP and GSNA and attenuated increases in HR. Microinjections of SHU9119 (a melanocortin 3/4 receptor antagonist) into the PVN in barodenervated rats attenuated increases in MAP, GSNA and HR elicited by the ARCN stimulation. ARCN neurons projecting to the PVN were immunoreactive for proopiomelanocortin, alpha-melanocyte stimulating hormone (alpha-MSH) and adrenocorticotropic hormone (ACTH). It was concluded that increases in MAP and GSNA and exaggeration of tachycardia elicited by the ARCN stimulation in barodenervated rats may be mediated via release of alpha-MSH and/or ACTH and glutamate from the ARCN neurons projecting to the PVN.

## Introduction

It is well established that different chemical phenotypes of neurons reside in the hypothalamic arcuate nucleus (ARCN) [1], [2]. Diverse cardiovascular responses are, therefore, expected from the chemical stimulation of the ARCN. Indeed, we have previously reported that chemical stimulation of the ARCN in the rats with intact baroreceptors elicited increases as well as decreases in mean arterial pressure (MAP) and sympathetic nerve activity (SNA) while there was always an increase in heart rate (HR) [3], [4]. The type of MAP response (i.e., decrease or increase) elicited from the ARCN depended on the level of baroreceptor activity which, in turn, was dependent on baseline MAP in rats with intact baroreceptors [4]. Thus, in rats with normal baroreceptor activation at normal baseline MAP, chemical stimulation of the ARCN elicited decreases in MAP and SNA and these responses were mediated via gamma aminobutyric acid type A (GABA-A), neuropeptide Y1 (NPY1) and opiate receptors in the hypothalamic paraventricular nucleus (PVN) [4]. This conclusion was based on our observations that blockade of GABA-A receptors (by gabazine), or NPY1 receptors (by BMS193885) or opiate receptors (by naloxone) in the PVN attenuated the depressor responses elicited by NMDA microinjections into the ipsilateral ARCN [4]. Moreover, combined blockade of these receptors in the PVN converted the depressor responses elicited by ARCN stimulation to pressor responses [4]. When the baseline MAP was decreased in these rats with intact baroreceptors by an intravenous infusion of sodium nitroprusside (SNP), increases (instead of decreases) in MAP and SNA were elicited by chemical stimulation of the ARCN [4]. The mechanism by which lowering of baseline MAP converted decreases to increases in MAP and SNA elicited from the ARCN is not known. It is well established that the ARCN includes neurons containing proopiomelanocortin (POMC) and cocaine- and amphetamine-regulated transcript (CART) [2]. POMC is the precursor for other peptides such as alpha-melanocyte stimulating hormone (alpha-MSH) and adrenocorticotropin (ACTH) [5]. POMC neurons have been reported to contain glutamate [6], [7]. ACTH, alpha-MSH and glutamate have been reported to increase the firing activity of neurons [8]–[11]. Based on this information, it was hypothesized that baroreceptor unloading may unmask the excitatory effects of these neurotransmitters (i.e., ACTH, alpha-MSH and glutamate) in the PVN and elicit pressor responses. Therefore, the effect of barodenervation was studied on the cardiovascular responses elicited by the chemical stimulation of the ARCN.

## Materials and Methods

### Ethics Statement

The NIH guide for “The Care and Use of Laboratory Animals, 7th Edition, 1996” was used for performing the experiments in this study. The protocols for the experiments were also approved by the Institutional Animal Care and Use Committee (IACUC) of UMDNJ-New Jersey Medical School, Newark, NJ, USA (Approval #: 11140D0215). Every effort was made to minimize the distress of the animals and to prevent their suffering.

### General Procedures

Adult male Wistar rats (Charles River Laboratories, Wilmington, MA, USA), weighing 300–360 g, were used in this study. The animals were housed in the animal care facility of this institution under controlled conditions with a 12-h light/dark cycle. Food and water were allowed to the animals ad libitum.

The details of general procedures used in this study have been published in our previous reports [12], [13]. Inhalation of isoflurane (2–3% in 100% oxygen) was used initially to anesthetize the rats. A cannula (PE 240) was placed in the trachea and the rats were artificially ventilated using a rodent ventilator (model 683; Harvard Apparatus, Holliston, MA, USA). The frequency and tidal volume were adjusted on the ventilator so that the end tidal CO_2_ was maintained at 3.5–4.5%. A cannula (PE 50) was placed in one of the femoral veins through which urethane (1 g/ml) was injected in 8–9 aliquots at 2-min intervals (total volume of the anesthetic solution was 0.4–0.45 ml injected over a period of about 16–18 min; using this procedure, a dose of 1.2–1.4 g/kg of urethane was used for anesthetizing the rats). Proper depth of anesthesia was confirmed by absence of a blood pressure (BP) increase and/or withdrawal of the limb in response to pinching of a hind paw. Tracheal administration of isoflurane was stopped when the administration of urethane was completed. A cannula (PE 50) was placed in one of the femoral arteries and connected to a pressure transducer (P23 XL-1, Argon Medical Devices, Clearwater, FL, USA). The pulsatile arterial pressure (PAP) signal was amplified (Model 1902, Cambridge Electronic Design Ltd, Cambridge, UK) (CED), digitized (Model micro-1401, CED, UK), and processed using Spike 2 software (CED, UK). MAP and HR were derived from PAP using Spike 2 software. A temperature controller (model TCAT-2AC, Physitemp Instruments, Clifton, NJ, USA) was used to monitor rectal temperature which was maintained at 37±0.5°C. All tracings were stored on a computer hard drive. After completion of the experiment, a high dose of urethane (2 g/kg, i.v.) was injected into the rats and an incision was made in one of the intercostal muscles to produce a pneumothorax; cessation of heart beat indicated that euthanasia was complete.

### Microinjection Technique

Anesthetized rats were fixed in a prone position in a stereotaxic instrument (David Kopf Instruments, Tajunga, CA, USA) with bite bar 3.3 mm below the interaural line. A small hole was drilled in the middle of the parietal bone just caudal to the bregma. Microinjections were delivered into the brain tissue using multi-barreled glass-micropipettes (tip size 20–40 µm). The micropipettes were inserted into the brain perpendicularly for microinjection into the rostral, middle and caudal regions of the ARCN (coordinates: 1.9–4.1 mm caudal to the bregma, 0.2–0.4 mm lateral to the midline, and 9.6–9.9 mm deep from the dura). When the protocol required microinjections into the PVN as well as the ARCN in the same experiment, the micropipettes were inserted into the PVN perpendicularly and the ARCN was approached using a micropipette at an 80° angle pointing rostrally. In these experiments, the coordinates for the PVN were: 1.6–1.9 mm caudal to the bregma, 0.3–0.5 mm lateral to the midline and 7.7–8.0 mm deep from the dura and coordinates for the ARCN were: 4.0–5.4 mm caudal to the bregma, 0.2–0.4 mm lateral to the midline, and 9.7–10.0 mm deep from the dura; using this approach, the tip of the micropipette reached the middle or caudal ARCN (coordinates: 2.7–4.1 mm caudal to the bregma, 0.2–0.4 mm lateral to the midline, and 9.6–9.9 mm deep from the dura). The ARCN and/or PVN were always identified by unilateral microinjections of N-methyl-D-aspartic acid (NMDA; 10 mM). The duration of microinjection was 10 sec. Controls for microinjections consisted of artificial cerebrospinal fluid (aCSF, pH 7.4). In order to avoid cardiovascular effects secondary to respiratory changes following ARCN stimulation, the rats were paralyzed with intravenous administration of pancuronium bromide (neuromuscular blocking agent; initial bolus injection of 1.2 mg/kg followed by 0.6 mg/kg bolus injections every 40 min). Appropriate depth of anesthesia was confirmed by pinching one of the hind paws before the administration of pancuronium. The volumes of all microinjections into the ARCN and PVN were 20 and 30 nl, respectively.

### Barodenervation

The carotid sinus, aortic depressor and recurrent laryngeal nerves were sectioned bilaterally for complete barodenervation. In the experiments where the cardiovascular responses to the ARCN stimulation were compared before and after barodenervation, silk sutures were placed loosely around these nerves bilaterally for the subsequent identification and sectioning of the nerves. Successful barodenervation was indicated by absence of reflex bradycardia or inhibition of greater splanchnic nerve activity in response to subsequent bolus injections of phenylephrine (PE, 10 µg/kg, i.v.). PE is a vasoconstrictor acting via alpha1-adrenoceptors located in arterioles.

### Greater Splanchnic Nerve Recording

A retroperitoneal approach was used to identify the greater splanchnic nerve (GSN) which was then sectioned at its junction with the celiac ganglion. The central end of the nerve was desheathed and placed on a bipolar silver wire hook electrode. Electrical activity of the whole GSN (GSNA) was amplified (×10,000–20,000), filtered (100–5000 Hz), digitized and stored on a computer hard drive. The digitized signals were full-wave rectified and integrated over consecutive 1 sec intervals using Spike 2 program (CED, UK). After completion of the experiment, the noise level of GSNA, determined by sectioning the GSN centrally, was subtracted from the GSNA amplitude.

### Retrograde Tracing of ARCN Projections and Immunohistochemistry

Aseptic conditions were used for surgical procedures in these experiments. The rats were anesthetized with pentobarbital sodium (50 mg/kg, i.p.) and fixed in a prone position in a stereotaxic instrument. Green retrobeads IX (original undiluted solution supplied by Lumafluor Inc.) were then microinjected (30 nl) into the PVN. After placing absorbable gelatin sponge (Surgifoam, Ethicon Inc., Somerville, NJ, USA) on the exposed brain surface, the skin over the wound was sutured. The rats were allowed to recover from the anesthesia and kept alive for a total of 7 days. An antibiotic (cefazolin, 30 mg/kg) was administered subcutaneously twice a day for 3 days and one dose of a slow release dosage form of an analgesic (buprenorphine SR, 1 mg/kg) was administered subcutaneously. The rats were again anesthetized with pentobarbital on the fifth day after the surgery and colchicine (120 µg, 10 µl) was microinjected into the lateral ventricle unilaterally (the coordinates for the lateral ventricle: 0.8–0.9 mm caudal to the bregma, 1.7–1.8 mm lateral to the midline, and 3.8–4.0 mm deep from the dura). After intracerebroventricular injection of colchicine, an antibiotic (cefazolin, 30 mg/kg, twice a day for 3 days) and an analgesic (one dose of buprenorphine SR, 1 mg/kg) were administered subcutaneously. Colchicine is known to inhibit axonal transport of neurons [14]; therefore, it was used to raise levels of peptides such as POMC, ACTH and alpha-MSH in the neuronal cell body and facilitate immunostaining for these peptides. The animals were then deeply anesthetized with pentobarbital (80 mg/kg, i.p.) on the seventh day and perfused first with heparinized normal saline which was followed by 2% paraformaldehyde solution containing 0.2% picric acid. The brains were removed and placed in 2% paraformaldehyde containing 0.2% picric acid for 48 hrs. On completion of the fixation procedure, one side of the brain surface was marked by a shallow cut and serial sections of the hypothalamic area were cut (40 µm) in a vibratome (1000 Plus Sectioning System, The Vibratome Company, St. Louis, MO, USA). The microinjection site of green retrobeads IX (Amax = 460 nm, Emax = 505 nm) and the retrogradely-labeled cells were visualized under a microscope (model AX70, Olympus Provis, Middlebush, NJ, USA). The sections were photographed (Neurolucida software, version 7.5, MicroBrightField Inc., Williston, VT, USA) and compared with a standard atlas [15]. The same sections containing the ARCN were then used for immunostaining of POMC, alpha-MSH and ACTH. The sections were rinsed (rinsing was always done 3 times, 10 min each) with 0.1 M phosphate buffered saline (PBS) and blocked for 60 min at room temperature with 10% normal goat serum (NGS) in 0.1 M PBS containing 0.3% Triton X-100 (TPBS). For POMC staining, the sections were incubated for 24 hours at 4°C with rabbit anti-POMC antibody (1∶5000; Phoenix Pharmaceuticals Inc; Burlingame, CA, USA; diluted with TPBS containing 3% NGS). After rinsing with PBS, the sections were incubated for 2 hours at 4°C with Cy3-goat anti-rabbit IgG (1∶200, Amax = 550 nm, Emax = 570 nm, Jackson Immuno-Research Laboratories Inc., West Grove, PA, USA; diluted with PBS containing 3% NGS). For ACTH and alpha-MSH staining, the same procedures were carried out except that rabbit anti-ACTH (1∶200; Phoenix Pharmaceuticals Inc.; diluted with TPBS containing 3% NGS) and rabbit anti-alpha-MSH antibody (1∶1000; Immunostar, Hudson, WI, USA; diluted with TPBS containing 2% NGS) were used as primary antibodies, respectively. After rinsing with PBS, the sections were incubated for 2 hours at 4°C with the same secondary antibody (Cy3-goat anti-rabbit IgG; 1∶200). After the completion of incubation with the primary and secondary antibodies in each of these procedures, the sections were rinsed in PBS, mounted on subbed slides, covered with Citifluor mountant medium (Ted Pella Inc., Redding, CA, USA) and coverslipped. The images of the sections were captured, 1 µm apart, using a laser scanning confocal microscope (AIR confocal microscope, Nikon Instruments Inc., Melville, NY, USA).

### Histological Identification of Microinjection Sites

The microinjection sites in the ARCN and PVN were marked by microinjections (20 and 30 nl, respectively) of diluted green retrobeads IX (1∶50). The animals were deeply anesthetized with urethane (2 g/kg, i.v.), perfused and fixed with 2% paraformaldehyde, serial sections of the hypothalamus were cut (40 µm) in a vibratome and mounted on slides. The microinjection sites were identified under a microscope (model AX70, Olympus Provis, Middlebush, NJ, USA). The sections were photographed (Neurolucida software, version 7.5, MicroBrightField Inc., Williston, VT, USA) and compared with a standard atlas [15].

### Drugs and Chemicals

The following drugs and chemicals were used: NMDA, NBQX disodium salt (2,3-dioxo-6-nitro-1,2,3,4-tetrahydrobenzo-[f]quinoxaline-7-sulfonamide disodium salt; a non-NMDA receptor antagonist), D-AP7 (D(-)-2-amino-7-phosphono-heptanoic acid; an NMDA receptor antagonist), SHU9119 (Ac-Nle-cyclo(-Asp-His-D-2-Nal-Arg-Trp-Lys)-NH2; a melanocortin (MC) 3/4 receptor antagonist), CART (55–102), green retrobeads IX, l-phenylephrine hydrochloride, pancuronium bromide, isoflurane, urethane, pentobarbital sodium, cefazolin and buprenorphine hydrochloride. All of the solutions for the microinjections were freshly prepared in aCSF. The composition of aCSF (pH 7.4) was as follows: NaCl (128 mM), KCl (3 mM), CaCl_2_ (1.2 mM), MgCl_2_ (0.8 mM), dextrose (3.4 mM) and HEPES (5 mM). Where applicable, the concentration of drugs refers to their salts. The vendors for different drugs and chemicals were as follows: NBQX, D-AP7 and SHU9119 (Tocris Bioscience, Ellisville, MO, USA), CART (55–102) (American Peptide Company, Sunnyvale, CA, USA), isoflurane (Baxter Pharmaceutical Products, Deerfield, IL, USA), pentobarbital (Ovation Pharmaceuticals Inc., Deerfield, IL, USA), cefazolin (West-ward Pharmaceutical Corporation, Eatontown, NJ, USA), buprenorphine (Hospira Inc., Lake Forest, IL, USA), green retrobeads IX (Lumafluor Inc., Durham, NC, USA). All other drugs and chemicals were obtained from Sigma-Aldrich Co. (St. Louis, MO, USA).

### Statistical Analyses

Maximum changes in MAP and HR in response to microinjections of different drugs were expressed as the means and standard error of the means (S.E.M.). Student's paired t-test was used for comparison of the following responses: increases in MAP and HR induced by the microinjections of NMDA into the ARCN before and after barodenervation, the microinjections of SHU9119 or the combined microinjections of NBQX and D-AP7 into the PVN. For analyses of the GSNA, the integrated signals obtained just before the microinjections of NMDA into the ARCN were averaged over a period of 60 sec. The integrated GSNA signals were averaged over a period of 60–90 sec when the responses to these treatments were maximal. The Student's paired t-test was used to compare the percentage changes in GSNA elicited by different treatments. The differences were considered to be significant at P<0.05.

## Results

In urethane-anesthetized rats, baseline values for MAP and HR were 94.6±2.7 mmHg and 444.4±5.5 bpm, respectively (n = 20).

### 1. ARCN Stimulation: Effect of Barodenervation

**Figure 1:**
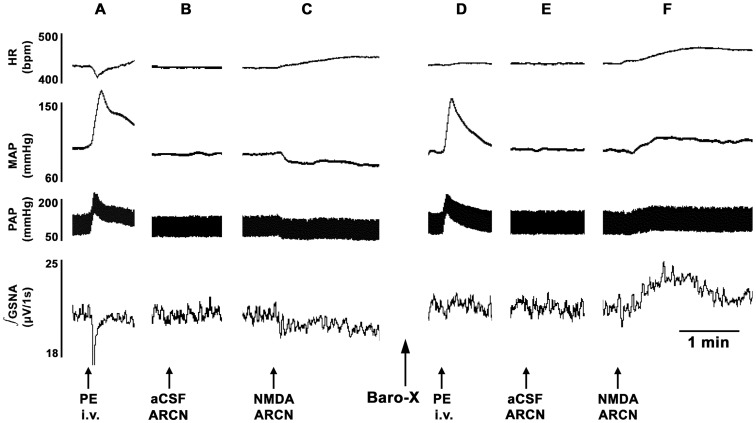
Tracings showing the effect of barodenervation on ARCN responses. Top trace: HR (beats/min), second trace: MAP (mmHg), third trace: PAP (mmHg), bottom trace: integrated GSNA (µV/1s). A: increase in MAP, reflex bradycardia and reflex inhibition of GSNA following a bolus injection of PE (10 µg/kg, i.v.). B: 3 min later, unilateral microinjection of aCSF into the ARCN elicited no changes in MAP, GSNA or HR. C: After an interval of 2 min, unilateral microinjection of NMDA (10 mM, 20 nl) into the ARCN elicited decreases in MAP and GSNA and an increase in HR. When effects of NMDA abated, barodenervation was performed; barodenervation increased baseline MAP, GSNA and HR (traces not shown). D: 60 min after bilateral barodenervation, when baseline MAP was completely recovered, bolus injection of the same concentration of PE elicited increases in MAP, but no reflex decreases in HR or GSNA confirming barodenervation was complete. E: After an interval of 3 min, aCSF was again microinjected into the ARCN; no changes in MAP, GSNA or HR were elicited. F: 2 min later, NMDA (10 mM, 20 nl) was again microinjected into the ARCN; increases (instead of decreases) in MAP and GSNA were elicited and tachycardic response was exaggerated. The following abbreviations are used in this and other figures. aCSF, artificial cerebrospinal fluid; ARCN, the hypothalamic arcuate nucleus; Baro-X, barodenervation; GSNA, greater splanchnic nerve activity; HR, heart rate (beats/min); MAP, mean arterial pressure (mmHg); NMDA, N-methyl-D-aspartic acid; PAP, pulsatile arterial pressure; PE, phenylephrine.

**Figure 2:**
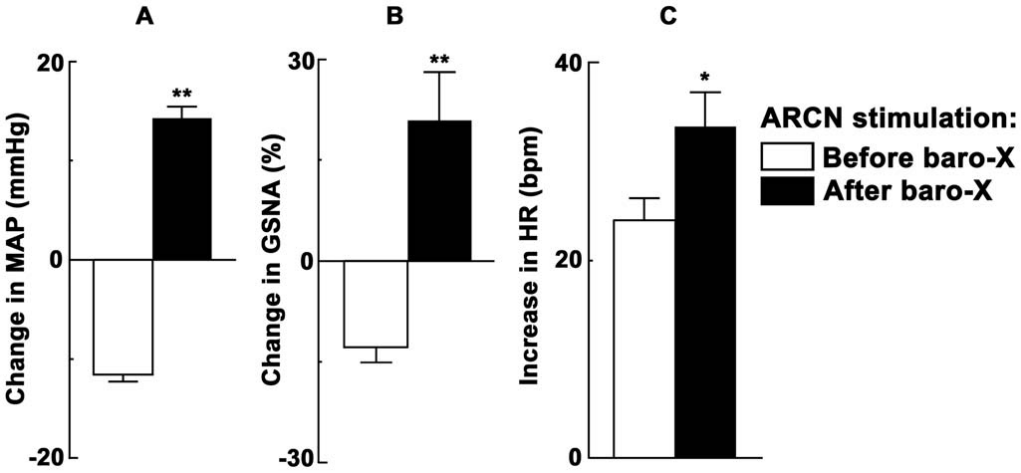
Group data showing the effect of barodenervation on ARCN responses. A: Microinjections of NMDA (10 mM, 20 nl) into the ARCN before barodenervation (open bars) elicited decreases in MAP (n = 5). Bilateral barodenervation elicited increases in baseline MAP, GSNA and HR (not shown) which lasted for 60 min. When baseline MAP was completely recovered, microinjections of NMDA (10 mM, 20 nl) into the ARCN after barodenervation (dark bars) elicited increases in MAP (**P<0.01). B: Decreases in GSNA elicited by microinjections of NMDA into the ARCN were converted to increases in GSNA by barodenervation (**P<0.01). C: Increases in HR elicited by the ARCN stimulation were exaggerated by barodenervation (*P<0.05).

In these experiments, a bolus injection of PE (10 µg/kg, i.v.) elicited increases in BP (MAP as well as pulsatile arterial pressure), reflex bradycardia and reflex inhibition of GSNA (Figure 1A). Three min later, unilateral microinjection of aCSF into the ARCN elicited no MAP, GSNA or HR responses (Figure 1B). Two min later, NMDA was microinjected into the ARCN: decreases in MAP and GSNA and increases in HR were elicited (Figure 1C). Twenty min later, when effects of NMDA subsided, bilateral barodenervation was done which elicited increases in baseline MAP, GSNA and HR. After an interval of 60 min, when baseline MAP was completely recovered, bolus injection of the same concentration of PE elicited increases in MAP, but no reflex bradycardia or inhibition of GSNA (Figure 1D). After an interval of 3 min, aCSF microinjected again into the same ARCN site elicited no MAP, GSNA or HR responses (Figure 1E). Two min later, NMDA microinjections were repeated at the same ARCN site; increases (instead of decreases) in MAP, GSNA were elicited and tachycardic responses were exaggerated (Figure 1F). Group data (n = 5) for these experiments are shown in Figure 2. The decrease in MAP in response to microinjections of NMDA into the ARCN before barodenervation was 11.6±0.7 mmHg. Bilateral barodenervation elicited increases in baseline MAP (32.0±3.6 mmHg), GSNA (48.6±9.4%) and HR (20.8±4.3 bpm). As mentioned earlier, after an interval of 60 min, when baseline MAP was completely recovered, bolus injection of the same concentration of PE elicited increases in MAP, but no reflex bradycardia or inhibition of GSNA. At this time, microinjection of NMDA into the ARCN elicited an increase in MAP (14.2±1.2 mmHg) which was significantly different from the decrease in MAP before the barodenervation (11.6±0.7 mmHg) (P<0.01, Figure 2A). Similar observations were made with GSNA; the decreases and increases in GSNA before and after the barodenervation were 12.8±2.3 and 20.9±7.0%, respectively (P<0.01, Figure 2B). Increases in HR elicited by microinjections of NMDA into the ARCN before and after barodenervation were 24.0±2.2 and 33.4±3.5 bpm, respectively (P<0.05, Figure 2C).

### 2. ARCN Stimulation: Effect of Ionotropic Glutamate Receptor (iGLUR) Blockade in the PVN in Barodenervated Rats

In these experiments, microinjections of NMDA were used to stimulate the middle or caudal ARCN and mixed solution (30 nl) of NBQX (non-NMDA receptor antagonist) and D-AP7 (NMDA receptor antagonist) was microinjected 20 min later into the ipsilateral PVN. The concentrations of NBQX (4 mM, 15 nl) and D-AP7 (10 mM, 15 nl) used were selected from our previous publications [13], [16].

**Figure 3:**
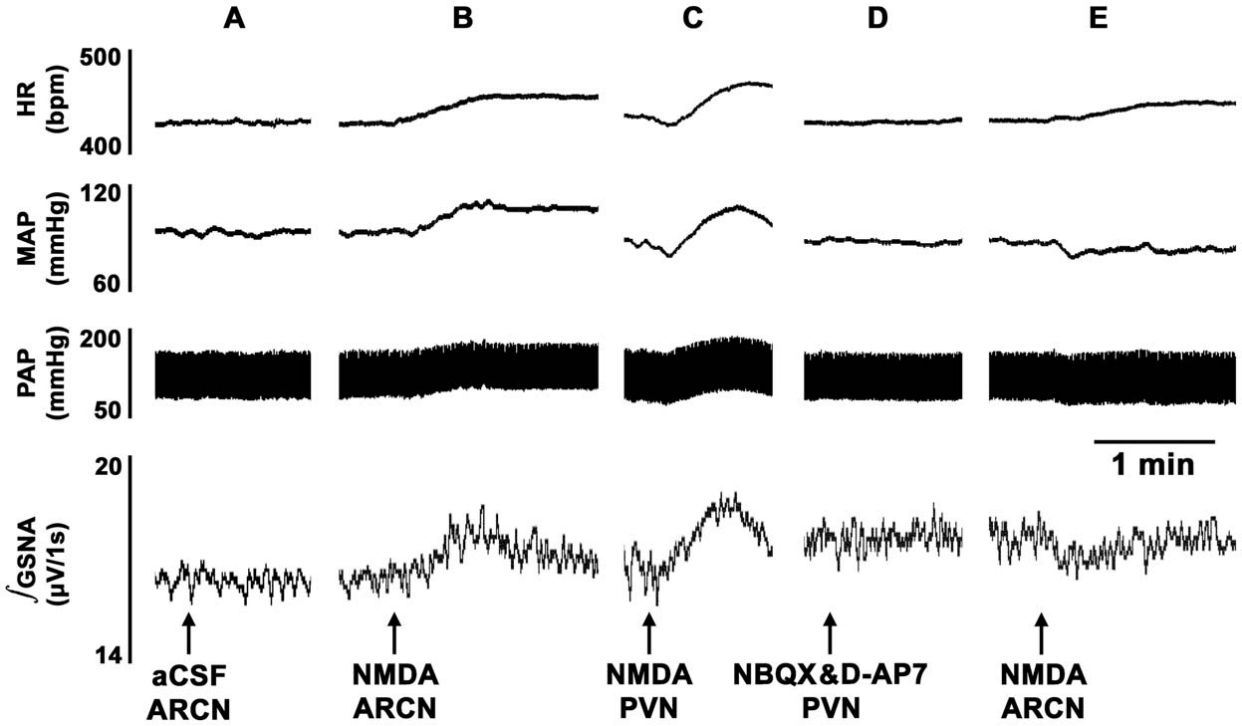
Tracings showing the effect of iGLUR blockade in the PVN on ARCN responses. A: In barodenervated rat, unilateral microinjection of aCSF into the ARCN elicited no changes in MAP, GSNA or HR. B: 2 min later, unilateral microinjection of NMDA (10 mM, 20 nl) into the ARCN elicited increases in MAP, GSNA and HR. C: 20 min later, ipsilateral PVN site was identified by a microinjection of NMDA (10 mM, 30 nl); increases in MAP, HR and GSNA were elicited. D: 20 min later, mixed solution (30 nl) of NBQX (4 mM, 15 nl) and D-AP7 (10 mM, 15 nl) was microinjected into the ipsilateral PVN; no changes in baseline MAP, HR or GSNA were elicited. E: After an interval of 3 min, NMDA (10 mM, 20 nl) was again microinjected into the ARCN; decreases (instead of increases) in MAP and GSNA were elicited and tachycardic response was attenuated. D-AP7 (NMDA receptor antagonist); NBQX (non-NMDA receptor antagonist); iGLURs, ionotropic glutamate receptors.

**Figure 4:**
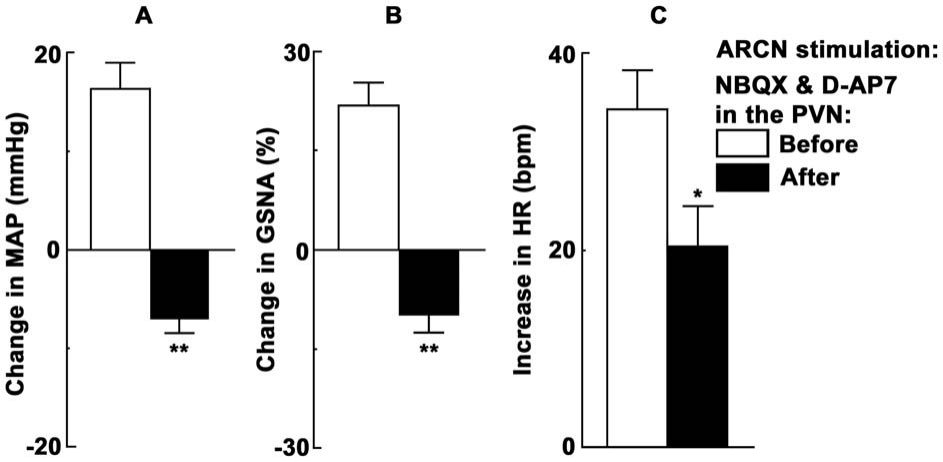
Group data showing the effect of iGLUR blockade in the PVN on ARCN responses. A: In barodenervated rats, before iGLUR blockade in the PVN (open bar), microinjections of NMDA (10 mM, 20 nl) into the ARCN elicited increases in MAP (n = 6). Combined microinjections of NBQX (4 mM, 15 nl) and D-AP7 (10 mM, 15 nl) into the ipsilateral PVN to block iGLURs elicited no changes in baseline MAP, GSNA or HR (not shown). Microinjections of NMDA (10 mM, 20 nl) into the ARCN after the blockade of iGLURs in the PVN (dark bar) elicited decreases in MAP (**P<0.01). B: Increases in GSNA elicited by microinjections of NMDA into the ARCN were converted to decreases in GSNA by the blockade of iGLUR in the PVN (**P<0.01). C: Increases in HR elicited by the ARCN stimulation were attenuated by the blockade of iGLUR in the PVN (*P<0.05).

In barodenervated rats, unilateral microinjection of aCSF into the ARCN elicited no MAP, GSNA or HR responses (Figure 3A). NMDA was microinjected into the ARCN: increases in MAP and GSNA and increases in HR were elicited (Figure 3B). Twenty min later, ipsilateral PVN site was identified by a microinjection of NMDA; increases in MAP, HR and GSNA were elicited (Figure 3C). After an interval of 20 min, mixed solution (30 nl) of NBQX (4 mM, 15 nl) and D-AP7 (10 mM, 15 nl) was microinjected into the ipsilateral PVN; no significant changes in baseline MAP, HR or GSNA were elicited (Figure 3D). After a gap of 3 min, NMDA microinjections were repeated at the same ARCN site: decreases (instead of increases) in MAP, GSNA were elicited and tachycardia was attenuated (Figure 3E). Group data (n = 6) for these experiments are shown in Figure 4. The increases and decreases in MAP in response to microinjections of NMDA into the ARCN before and after microinjections of NBQX and D-AP7 into the PVN were 16.3±2.6 and 7.0±1.5 mmHg, respectively (P<0.01, Figure 4A). Similarly, the increases and decreases in GSNA were 21.8±3.4 and 9.8±2.8%, respectively (P<0.01, Figure 4B). Increases in HR elicited by microinjections of NMDA into the ARCN before and after microinjections of NBQX and D-AP7 into the PVN were 34.2±4.0 and 20.5±3.9 bpm, respectively (P<0.05, Figure 4C).

### 3. ARCN Stimulation: Effect of MC Receptor Blockade in the PVN

We have previously reported [4] that in non-barodenervated rats with normal baseline MAP, the depressor and tachycardic responses to microinjections of NMDA into the ARCN were not altered by microinjections of SHU9119 (MC 3/4 receptor antagonist; 2 mM) into the ipsilateral PVN.

**Figure 5:**
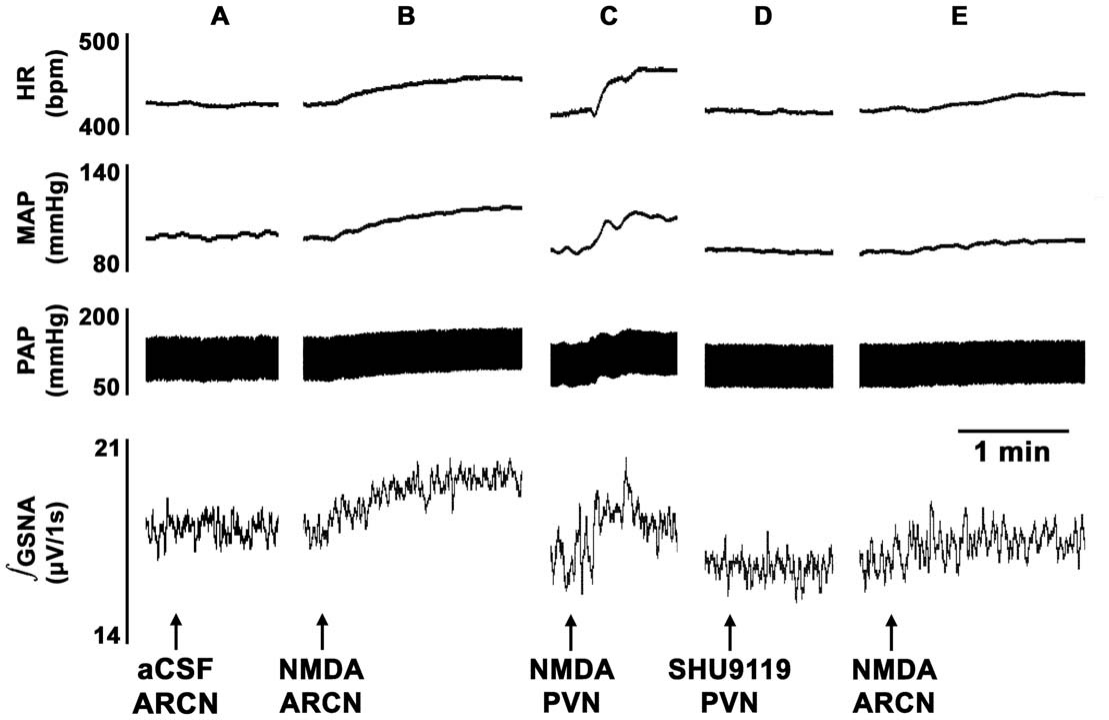
Tracings showing the effect of melanocortin receptor blockade in the PVN on ARCN responses. A: In barodenervated rat, unilateral microinjection of aCSF into the ARCN elicited no changes in MAP, GSNA or HR. B: 2 min later, unilateral microinjection of NMDA (10 mM, 20 nl) into the ARCN elicited increases in MAP, GSNA and HR. C: 20 min later, ipsilateral PVN site was identified by a microinjection of NMDA (10 mM, 30 nl); increases in MAP, HR and GSNA were elicited. D: 20 min later, SHU9119 (2 mM, 30 nl) was microinjected into the ipsilateral PVN; no changes in baseline MAP, HR or GSNA were elicited. E: After an interval of 3 min, NMDA (10 mM, 20 nl) was again microinjected into the ARCN; increases in MAP, GSNA and HR were attenuated. SHU9119 (melanocortin 3/4 receptor antagonist).

**Figure 6:**
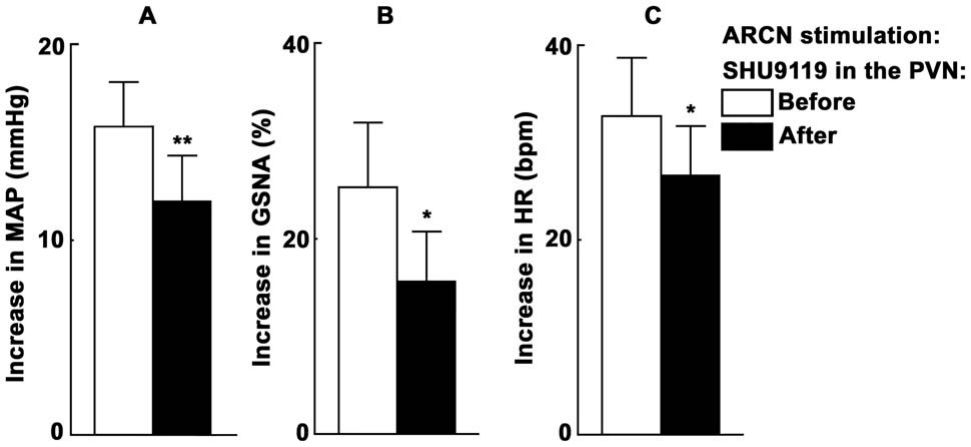
Group data showing the effect of melanocortin receptor blockade in the PVN on ARCN responses. A: In barodenervated rats, microinjections of NMDA (10 mM, 20 nl) into the ARCN before the blockade of MC 3/4 receptors in the ipsilateral PVN by SHU9119 (open bar) elicited increases in MAP (n = 5). Microinjections of SHU9119 (2 mM, 30 nl) into the ipsilateral PVN elicited no changes in baseline MAP, GSNA or HR (not shown). Microinjections of NMDA (10 mM, 20 nl) into the ARCN after the blockade of MC 3/4 receptors in the ipsilateral PVN (dark bar) elicited smaller increases in MAP than those before microinjections of SHU9119 (**P<0.01). B: Increases in GSNA elicited by microinjections of NMDA into the ARCN were attenuated by the blockade of melanocortin 3/4 receptors in the PVN (*P<0.05). C: Increases in HR elicited by the ARCN stimulation were attenuated by the blockade of melanocortin 3/4 receptors in the PVN (*P<0.05).

In barodenervated rats, however, increases in MAP, GSNA and HR elicited by microinjections of NMDA into the ARCN were attenuated by prior microinjections of the same concentration of SHU9119 into the ipsilateral PVN. Unilateral microinjection of aCSF into the ARCN elicited no MAP, GSNA or HR responses (Figure 5A). Microinjection of NMDA into the ARCN elicited increases in MAP, GSNA and HR (Figure 5B). Twenty min later, the ipsilateral PVN was identified by microinjection of NMDA (Figurer 5C). After a gap of 20 min, SHU9119 (2 mM) was microinjected into the PVN; no significant changes in the baseline MAP, GSNA or HR were observed (Figure 5D). Three min later, NMDA microinjection was repeated into the ARCN; the increases in MAP, GSNA and HR were attenuated (Figure 5E). Group data (n = 5) for this experiment are shown in Figure 6. The increases in MAP in response to NMDA in the ARCN before and after the microinjections of SHU9119 into the PVN were 15.8±2.9 and 12.0±2.3 mmHg, respectively (P<0.01, Figure 6A). Similarly, the increases in GSNA were 25.2±6.6 and 15.7±5.1%, respectively (P<0.05, Figure 6B). The increases in HR were 32.6±6.1 and 26.8±4.9 bpm, respectively (P<0.05, Figure 6C). In this group of rats, the microinjections of SHU9119 into the PVN did not alter any cardiovascular responses to the microinjection of the NMDA at the same site (i.e., the PVN) (data not shown).

### 4. Effect of CART (55–102) in the PVN

Most of POMC containing neurons in the ARCN co-express CART [17]. Therefore, it is possible that CART may also be released in the PVN following the ARCN stimulation. Cardiovascular responses to microinjections of CART into the PVN were, therefore, tested in barodenervated rats (n = 4). Microinjections of CART (55–102) (0.8 mM) into the PVN elicited no BP or HR responses. The concentration of CART (55–102) (0.8 mM) used in these experiments, was selected from published literature [18].

### 5. POMC, Alpha-MSH and ACTH Immunoreactive ARCN Cells Retrogradely Labeled from the PVN

**Figure 7:**
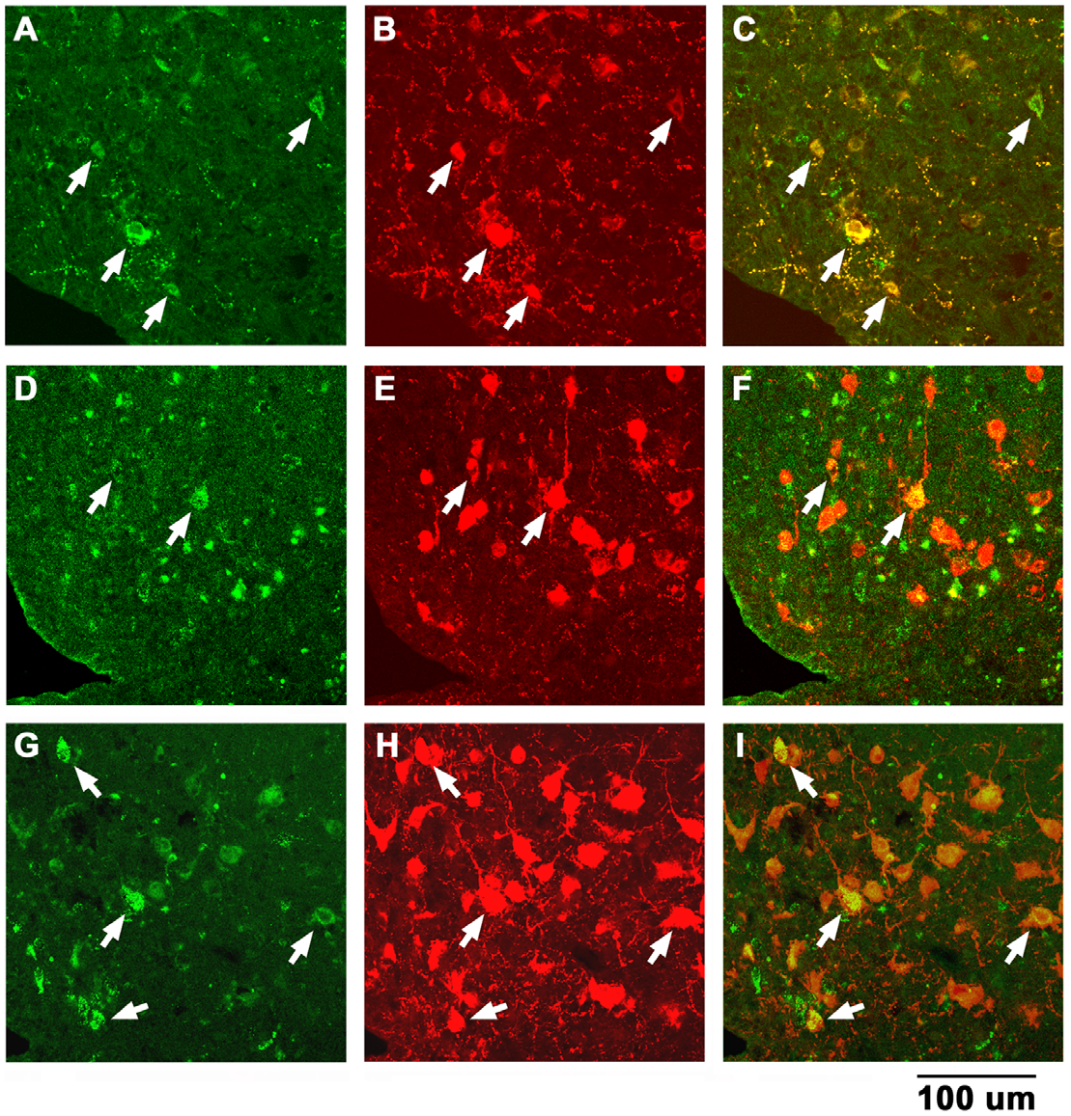
Identification of POMC, alpha-MSH and ACTH immunoreactive ARCN cells retrogradely labeled from the PVN. A: Microinjection (30 nl) of green retrobeads IX into the PVN retrogradely labeled neurons in the ipsilateral ARCN. B: POMC immunoreactive neurons in the section shown in A. C: Some retrogradely labeled cells in the ARCN contained POMC (white arrows) as indicated by the merged images of A and B. D: ARCN neurons retrogradely labeled from PVN (different rat). E: alpha-MSH immunoreactive neurons in the section shown in D. F: The merged images of D and E indicated that some retrogradely labeled cells in the ARCN contained alpha-MSH (white arrows). G: ARCN neurons retrogradely labeled from PVN (different rat). H: ACTH immunoreactive neurons in the section shown in G. I: The merged images of G and H indicated that some retrogradely labeled cells in the ARCN contained ACTH (white arrows). Abbreviations: POMC, proopiomelanocortin; alpha-MSH, alpha-melanocyte stimulating hormone; ACTH, adrenocorticotropic hormone.

ARCN neurons were retrogradely labeled by microinjection of green retrobeads IX into the ipsilateral PVN (Figure 7, A, D and G). POMC, alpha-MSH and ACTH immunoreactive neurons in the same sections (i.e., Figure 7, A, D and G) are shown in Figure 7, B, E and H, respectively. Some retrogradely labeled cells in the ARCN contained POMC (Figure 7C), alpha-MSH (Figure 7F) and ACTH (Figure 7I) as revealed by the merged images.

### 6. Identification of Microinjection Sites

**Figure 8:**
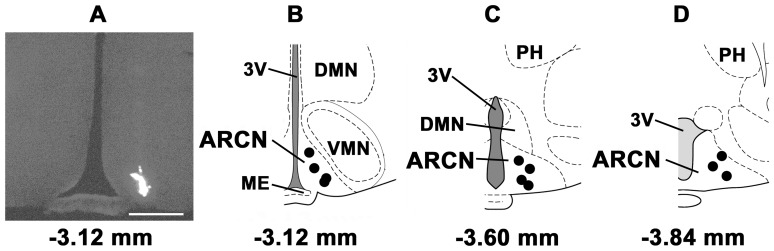
Histological identification of microinjection sites in the ARCN. A: Microinjection site in the ARCN marked with green retrobeads IX (20 nl) (a typical site). B–D: Composite diagrams of ARCN sections at levels 3.12 mm, 3.60 mm and 3.84 mm caudal to the bregma showing microinjection sites (n = 11). Each dark spot represents one microinjection site in one animal. Calibration bar in panel A = 500 µm. Abbreviations: DMN, the hypothalamic dorsomedial nucleus; ME, the hypothalamic median eminence; PH, the posterior hypothalamic nucleus; VMN, the hypothalamic ventromedial nucleus; 3V, the third ventricle.

**Figure 9:**
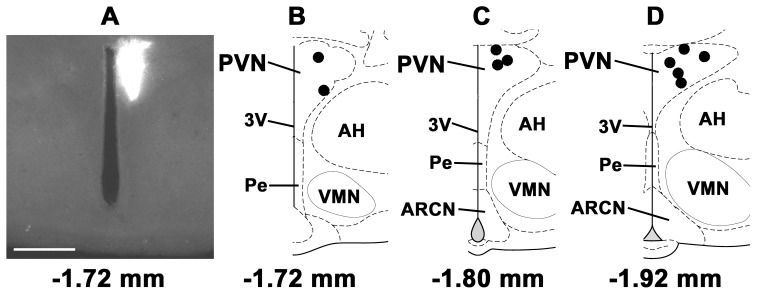
Histological identification of microinjection sites in the PVN. A: A typical microinjection site in the PVN marked with green retrobeads IX (30 nl). B-D: Composite diagrams of PVN sections at levels 1.72 mm, 1.80 mm, 1.92 mm caudal to the bregma showing microinjection sites (n = 10). Each dark spot represents one microinjection site in one animal. Calibration bar in panel A = 500 µm. Abbreviations: AH, the anterior hypothalamic area; Pe, the hypothalamic periventricular nucleus.

A typical ARCN microinjection site (marked by diluted green retrobeads IX) is shown in Figure 8A. Composite diagrams of the ARCN microinjection sites are shown in Figure 8, B–D (n = 11). A typical microinjection site in the PVN is shown in Figure 9A. The composite diagrams of PVN microinjection sites (n = 10) are presented in Figure 9, B–D. The volumes of green retrobeads IX microinjected into the ARCN and the PVN were 20 and 30 nl, respectively.

## Discussion

New observations made in this study are as follows: 1) bilateral barodenervation converted decreases in MAP and GSNA elicited by the ARCN stimulation to increases in MAP and GSNA and exaggerated increases in HR, 2) blockade of iGLURs in the PVN in barodenervated rats converted increases in MAP and GSNA elicited by the ARCN stimulation to decreases in MAP and GSNA and attenuated increases in HR, and 3) blockade of MC 3/4 receptors in the PVN in barodenervated rats attenuated increases in MAP, GSNA and HR elicited by the ARCN stimulation. It was concluded that increases in MAP and GSNA and exaggeration of tachycardia elicited by the ARCN stimulation in barodenervated rats may be mediated via release of alpha-MSH and/or ACTH and glutamate from the ARCN neurons projecting to the PVN.

Our results showed that the type of BP response (depressor or pressor) elicited by the chemical stimulation of the ARCN depended on the level of baroreflex activity. For example, we have reported that in rats with intact baroreceptors, microinjections of NMDA into the ARCN elicited depressor responses at normal baseline BP [4]. When the BP was lowered in these rats by infusion of SNP, pressor responses were elicited by chemical stimulation of the ARCN [4]. This observation prompted us to test if the effects of baroreceptor unloading were responsible for the conversion of depressor responses at normal baseline BP to pressor responses at lower levels of BP. In the present study, experiments were carried out in barodenervated rats. Acute barodenervation resulted in increases in MAP which returned to basal levels within 60 min. The recovery of MAP after barodenervation has been explained by the failure to maintain a sustained elevation of sympathetic activity in these rats [19]. At this time, chemical stimulation of the ARCN elicited increases in MAP. The mechanism of conversion of decreases in MAP elicited by ARCN stimulation in rats with intact baroreceptors to increases in these variables in barodenervated rats can be explained as follows. Barodenervation resulted in abolition of excitatory inputs from baroreceptor afferents to the nucleus tractus solitarius (NTS) neurons involved in this reflex. As a result, the activity of excitatory glutamatergic inputs from the NTS to the caudal ventrolateral medullary depressor area (CVLM) decreased, the activity of inhibitory GABAergic inputs from the CVLM to the rostral ventrolateral medullary pressor area (RVLM) decreased, and the excitability of presympathetic RVLM neurons increased following this disinhibition [20]–[23]. Under these conditions (e.g., increased excitability of RVLM neurons due to barodenervation), the responses to activation of excitatory ARCN neurons (e.g., ACTH, glutamate and alpha-MSH neurons) by microinjections of NMDA into the ARCN are expected to be expressed more readily. Thus, release of excitatory neurotransmitters in the PVN following the ARCN stimulation after barodenervation is expected to elicit pressor responses more readily. In this context, it may be noted that ACTH, alpha-MSH and glutamate have been reported to increase the firing activity of neurons [8]–[11]. Chemical stimulation of the PVN has been reported to excite spinally projecting RVLM neurons [24]–[26]. Activation of the projection from the PVN to the RVLM has been reported to elicit increases in BP and renal sympathetic nerve activity via the release of glutamate in the intermediolateral cell column of the spinal cord (IML) [27]. The ARCN includes neurons containing inhibitory neurotransmitters (e.g., NPY, GABA and beta-endorphin neurons) [5], [28]–[32]. As mentioned earlier, chemical stimulation of the ARCN in rats with intact baroreceptors elicited depressor responses at normal baseline MAP and this response was mediated via GABA-A, NPY1 and opiate receptors in the PVN [4]. Although microinjections of NMDA into the ARCN of barodenervated rats are expected to simultaneously activate neurons containing inhibitory neurotransmitters (e.g., GABA, NPY and beta-endorphin), depressor responses were not manifested by release of these inhibitory neurotransmitters in the PVN and pressor responses predominated. This effect could be attributed to increased excitability of RVLM neurons in barodenervated rats as mentioned earlier.

Chemical stimulation of the ARCN always elicited tachycardia [3], [4]. The tachycardic responses elicited by the ARCN stimulation were exaggerated by barodenervation. We have previously reported that the increases in HR elicited from the ARCN are mediated via the activation of spinal cord iGLURs [3]. It is known that glutamatergic inputs from the RVLM activate the sympathetic preganglionic neurons in the IML [33]. Thus, increased excitability of the RVLM may result in exaggeration of tachycardic responses elicited by the chemical stimulation of the ARCN. Recall that cardiovascular responses to ARCN stimulation are mediated via the PVN [4].

Vagal mechanism may also be involved in the exaggeration of tachycardic responses to the stimulation of ARCN-PVN pathway. Decrease in vagal activity to the heart has been implicated in the tachycardic responses elicited by ARCN stimulation [3]. We have also reported that tachycardic responses elicited by microinjection of NMDA into the PVN were mediated via inhibition of vagal activity [34]. According to this report [34], stimulation of the PVN activates glutamatergic inputs to GABAergic interneurons in the NTS, which, in turn, inhibit glutamatergic neurons projecting to the nucleus ambiguus (nAmb). Decrease in the activity of glutamatergic inputs to the nAmb neurons results in tachycardia. On the other hand, baroreceptor inputs excite the glutamatergic inputs to the nAmb neurons, eliciting bradycardia. Thus, activations of ARCN-PVN pathway and baroreceptor afferents have opposite effects on HR; the former elicits tachycardia while the latter elicits bradycardia. Barodenervation abolishes the excitatory inputs to the NTS neurons and decreases the activity of glutamatergic inputs to the nAmb neurons. Barodenervation-induced abolition of the mechanism mediating bradycardia results in exaggeration of tachycardic responses elicited by the activation of ARCN-PVN pathway.

In this paper, we have provided evidence that increases in MAP and GSNA elicited by ARCN stimulation are mediated via iGLURs in the PVN. This conclusion is based on our results that blockade of iGLURs in the PVN (by microinjections of D-AP7 and NBQX) converted the increases in MAP, and GSNA elicited by ARCN stimulation to decreases in these variables. It is known that subpopulations of POMC-containing neurons in the ARCN are glutamatergic [35], [36] and numerous glutamate-immunoreactive synapses [37], [38] and NMDA receptor mRNA [39] are present in the PVN. Thus, release of an excitatory amino acid (probably glutamate) in the PVN may directly or indirectly participate in the increases in MAP, GSNA and HR elicited by ARCN stimulation in barodenervated rats.

Immunohistochemical experiments in this study, in agreement with previous reports [36], [40], revealed the presence of POMC neurons in the ARCN. POMC is a precursor of both ACTH and alpha-MSH. Therefore, POMC neurons in the ARCN may release alpha-MSH and/or ACTH at their terminals in the PVN. Alpha-MSH has been reported to cause depolarization of PVN neurons and increase their firing activity [11]. We have previously reported microinjections of ACTH increased in BP, GSNA and HR in other brain areas (e.g., the RVLM) and these responses were completely blocked by prior microinjections of SHU9119 at the same site [12]. Thus, chemical stimulation of the ARCN may activate POMC neurons which results in the release of alpha-MSH/ACTH in the PVN. Excitation of PVN neurons, in turn, results in increases in MAP, GSNA and HR.

Some POMC neurons co-express CART [17]. The release of CART, if any, in the PVN is expected to stimulate PVN neurons based on the information that it has stimulatory effects on neurons [41]. However, it is unlikely that release of CART in the PVN was involved in mediating the increases in MAP, GSNA and HR elicited by ARCN stimulation because microinjections of this peptide into the PVN failed to elicit any cardiovascular response. The concentration of CART injected into the PVN was selected based on reports in which intracisternal administration of CART elicited pressor responses [18].

### Conclusion and Significance

As mentioned earlier, the main conclusion in this study is that stimulation of the ARCN in barodenervated rats elicits increases in BP, HR and SNA. ARCN neurons have also been reported to be activated by stress [42], [43]. Thus, ARCN may be one of the sites via which stress is likely to increase BP, HR, and SNA. These stress-induced cardiovascular effects are likely to be more pronounced when the baroreceptor function is compromised. Indeed, cardiovascular responses to stress have been reported to be exaggerated in disease conditions such as hypertension and obesity [44]–[47]. In this context, it may be noted that baroreceptor function is known to be attenuated in hypertension [48]–[51] and obesity [52], [53]. The results of the present study may provide a new line of thought regarding the mechanisms responsible for abnormal cardiovascular function in diseases such as hypertension and obesity.
